# Turnaround Time of Laboratory Investigations and Factors Responsible for Delays in a Tertiary Care Teaching Hospital in Eastern India: A Cross-Sectional Study

**DOI:** 10.7759/cureus.100703

**Published:** 2026-01-03

**Authors:** Naveen K G, Shamshad Ahmad, Pragya Kumar, Mala Mahto, Shreekant Bharti

**Affiliations:** 1 Community Medicine, Dhanalakshmi Srinivasan Institute of Medical Sciences and Hospital, Perambalur, IND; 2 Community and Family Medicine, All India Institute of Medical Sciences, Patna, Patna, IND; 3 Biochemistry, All India Institute of Medical Sciences, Patna, Patna, IND; 4 Pathology and Laboratory Medicine, All India Institute of Medical Sciences, Patna, Patna, IND

**Keywords:** hospital information system (his), laboratory efficiency, staff awareness, timeliness, turnaround time (tat)

## Abstract

Background

The timely delivery of laboratory reports is crucial for guiding medical decisions and ensuring optimal patient care. The efficiency of clinical laboratory services, measured by turnaround time (TAT), directly impacts patient outcomes. This study aimed to evaluate the TAT for biochemical and haematological investigations at AIIMS Patna, to identify areas for improvement.

Methods

A descriptive cross-sectional study using Hospital Information System (HIS) data from January to March 2022 was undertaken. Only samples with same-day requisition and collection were included to avoid patient-related delays. A total of 201,552 analysable samples - 178,596 biochemistry (88.6%) and 22,956 haematology (11.4%) - were extracted. Time stamps for requisition, sample acceptance, result entry, and result validation were retrieved. TAT was quantified as pre-analytical (requisition to sample acceptance), analytical (acceptance to result entry), post-analytical (entry to validation), and total TAT. Median values, with 10th to 90th percentiles, were calculated. Additionally, awareness of TAT was assessed among 46 laboratory staff using a structured questionnaire.

Results

Of 521,822 laboratory records retrieved from the HIS, 201,552 samples (38.6%) with complete and valid time-stamp data were available for analysis, after exclusion of records with implausible or negative time intervals. The overall median total TAT was 278 minutes (4 hours 38 minutes), with comparable median values for biochemistry (279 minutes) and haematology (273 minutes). Phase-wise analysis showed that the pre-analytical phase contributed approximately 56%, and the post-analytical phase 18%, of the total TAT, indicating that non-analytical processes accounted for nearly three-quarters of the overall delay. When analytical benchmarks were applied, 136,741 biochemistry samples (76.5%) met the <90-minute criterion, and 16,305 haematology samples (71.1%) met the <60-minute criterion. Operational factors contributing to delays were identified across outpatient and inpatient settings. The staff awareness assessment included 46 laboratory workers, 36 of whom were laboratory technicians (78.3%), and 10 of whom were phlebotomists (21.7%). Of these, 23 (50%) showed that they were aware of TAT.

Conclusion

Evaluation of more than 200,000 investigations showed satisfactory analytical performance, but highlighted significant pre- and post-analytical delays, prolonging overall TAT. Strengthening HIS data capture, addressing workflow bottlenecks, and improving staff awareness are essential for optimising laboratory efficiency. The study establishes baseline TAT metrics for continuous quality improvement and enhanced patient care at AIIMS Patna.

## Introduction

The rise in patients seeking public health services in developing nations illustrates the importance of efficient, cost-effective healthcare delivery [1,2]. The speed at which healthcare providers diagnose and prioritise treatment is a key indicator of their efficiency [3]. Prompt clinical laboratory services are crucial for guiding medical decisions and patient care, as laboratory results are fundamental to many medical judgements [4]. Quick access to lab findings significantly impacts patient outcomes and diagnostic performance [5]. Delays in lab results frustrate patients and hinder timely clinical decisions [5,6]. While labs prioritise accuracy and reliability, timely reporting is often neglected [7]. However, timely and precise reports enable early diagnosis, appropriate treatment, and improved patient outcomes, thereby reducing healthcare costs associated with extended hospital stays [5,8-10].

Turnaround time (TAT) in healthcare refers to the duration from test requisition to when the report reaches the physician. TAT definitions vary based on the specific test (e.g., haemoglobin and potassium), patient type (e.g., emergency department and ICU), priority level (e.g., stat, urgent, and routine), and the scope of activities included (e.g., from order time or sample receipt in the lab) [5]. Definitions can differ between laboratories [5,11,12].

Clinicians typically perceive TAT as the duration between test requisition and access to results [13]. Laboratories, however, often define TAT more narrowly by excluding components beyond their direct control, such as phlebotomy and specimen transport [14,15]. As a result, laboratory performance assessments frequently focus on intra-laboratory TAT, defined as the interval from sample registration to result authorisation [5]. While this approach simplifies measurement, it fails to capture delays occurring across the broader “brain-to-brain loop,” or total test cycle, which encompasses pre-analytical, analytical, and post-analytical phases, and reflects the actual experience of clinicians and patients [16].

Assessing and improving TAT is a critical component of laboratory quality management [17]. ISO 15189:2012 mandates that clinical laboratories establish, monitor, and periodically evaluate TATs, in consultation with service users, to ensure timely and reliable diagnostic support [18]. Prolonged TAT has been associated with increased repeat testing, duplication of investigations, delayed clinical decision-making, and higher healthcare costs, underscoring the importance of efficient laboratory workflows for optimal patient management [19,20]. Despite these international recommendations, limited evidence exists from resource-constrained public-sector hospitals in low- and middle-income countries. In particular, there is a paucity of studies that comprehensively evaluate TAT across the entire testing cycle, using routinely generated Hospital Information System (HIS) data, and that simultaneously examine workflow- and system-level contributors to delay in both outpatient and inpatient settings. Furthermore, institution-specific evidence integrating objective HIS-based TAT measurement with stakeholder perspectives on operational bottlenecks remains scarce.

Accordingly, the present study was undertaken to quantitatively assess total TAT for routine haematology and biochemistry investigations at a tertiary-care public hospital, using HIS-derived time stamps and encompassing the pre-analytical, analytical, and post-analytical phases. In addition, the study aimed to systematically identify operational and workflow-related factors influencing TAT in outpatient and inpatient settings, and to evaluate awareness regarding TAT among central laboratory personnel at the AIIMS Patna.

## Materials and methods

This hospital-laboratory-based cross-sectional study was conducted exclusively at the All India Institute of Medical Sciences, Patna, India, an institute of national importance (INI), established in 2012 under the Pradhan Mantri Swasthya Suraksha Yojana (PMSSY), with a 960-bed capacity and 42 specialised departments, providing comprehensive outpatient, inpatient, and emergency services to Bihar and neighbouring states. The central laboratory is equipped with advanced automated systems, including the Beckman Coulter AU5800 series, ADVIA Centaur® XPT Immunoassay System, STA Compact Max3, Sysmex XN-1000™ Haematology Analyzer, and ROLLER 20LC, enabling high-throughput and precise biochemical and haematological testing. Although the central laboratory comprises biochemistry, microbiology, and pathology departments, the present paper focuses exclusively on routine investigations performed by the departments of biochemistry and haematology, which together constitute a major component of the laboratory’s routine workload. AIIMS Patna operates through a fully digitised HIS, allowing a paper-free workflow. The study sought to examine the TATs of biochemical and haematological samples, to pinpoint potential areas for enhancement.

**Figure 1:**
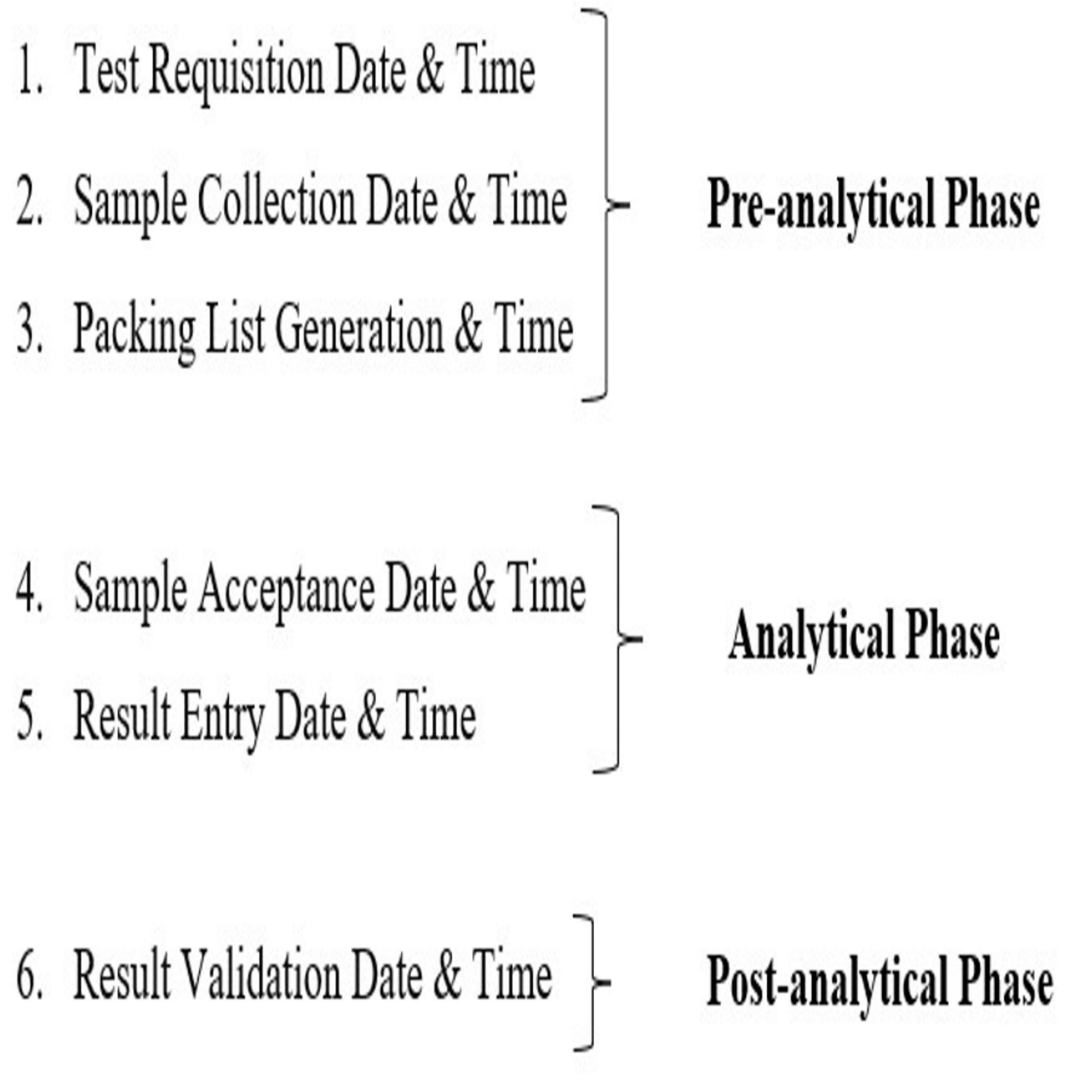
Time stamps retrieved from Hospital Information System (HIS) Created by the authors using Microsoft Word 2010 (Microsoft® Corp., Redmond, WA, USA).

**Figure 2:**
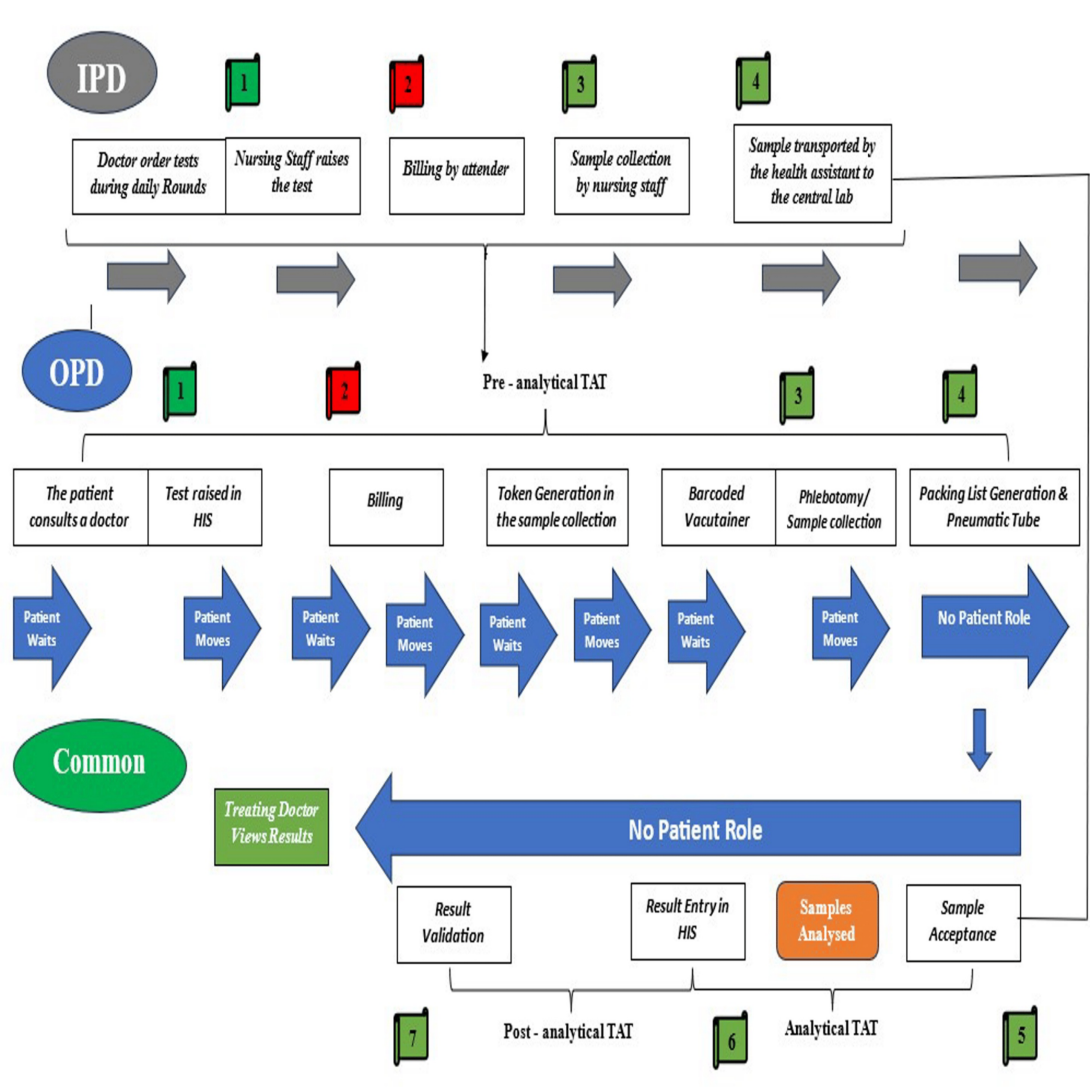
OPD and IPD event flow in the testing process at AIIMS Patna Created by the authors using Microsoft Word 2010 (Microsoft® Corp., Redmond, WA, USA). OPD, outpatient department; IPD, inpatient department; TAT, turnaround time; HIS, hospital information system

For the estimation of laboratory TAT, predefined time stamps routinely recorded in the HIS were utilised. These system-generated time stamps correspond to sequential milestones across the pre-analytical, analytical, and post-analytical phases of laboratory testing. Figure 1 shows the specific time points that were recorded in the HIS, and how they were grouped by phase. Figure 2 shows the overall sample testing workflow and process flow that connects these time stamps for both outpatient and inpatient samples. Expert consensus from the Departments of Haematology and Biochemistry selected the investigations for the study, and Appendix A provides detailed information. Timestamp data for all eligible routine haematology and biochemistry investigations were retrospectively extracted for the period January to March 2022. A universal sampling approach was adopted, whereby all samples corresponding to expert-specified routine investigations, with complete and valid timestamp data, were included in the analysis.

The above-mentioned sample testing process (Figure 2) reflects the locally established laboratory workflow at the study centre. In the outpatient setting, the pre-analytical sequence includes registration, billing, token generation, sample collection, barcode assignment, and dispatch of samples to the central laboratory through a pneumatic tube system. In contrast, for inpatient samples, test requisition and sample collection are performed in the wards, with samples transported physically to the central laboratory by health assistants. Following receipt in the laboratory, samples from both settings converge into a common analytical pathway, comprising sample acceptance, analysis, and electronic result entry into the HIS, followed by post-analytical steps, including result validation and clinician access to reports. TAT assessment was conducted within the context of these institution-specific operational practices, recognising that laboratory workflows and process configurations may differ across healthcare settings.

Due to technical limitations within the HIS, billing date and time could not be retrieved and were therefore excluded from the TAT assessment. In addition, the retrieved HIS time-stamp data did not permit differentiation between outpatient and inpatient samples at the level of individual laboratory records. Records with implausible or negative time intervals, arising from inconsistencies in system-generated time stamps, were excluded from analysis, as such values do not reflect a valid chronological sequence of laboratory processes. Exclusion of these records may have introduced selection bias if timestamp inconsistencies were not randomly distributed across samples or workflow stages; however, the exclusion was considered necessary to preserve the interpretability of TAT estimates. These constraints were recognised a priori as limitations of the study; nevertheless, identification of such gaps in routinely captured system data forms an integral part of evaluating existing laboratory workflows and highlights operational areas that may warrant further review and system-level refinement.

Only samples for which test requisition and sample collection occurred on the same calendar day were included. This restriction was applied to exclude situations, particularly in the outpatient setting, where test requisition is generated by clerical staff at the Outpatient Department (OPD), but patients may defer sample collection to a subsequent day, based on convenience, introducing delays unrelated to the sample testing cycle (both extra- and intra-laboratory), and thereby inflating TAT estimates. An activity-wise description of laboratory processes corresponding to individual time stamps is provided in Appendix B. 

Total TAT was operationally defined as the interval between test requisition and result validation. Pre-analytical TAT was defined as the time from requisition to sample acceptance in the central laboratory, analytical TAT as the duration between sample acceptance and result entry into the HIS, and post-analytical TAT as the interval between result entry and validation. Data analysis was performed using Jamovi Solid Version 2.6.44 (The Jamovi Project, Sydney, Australia). As the primary objective was to describe TAT patterns rather than to test hypotheses, analyses were restricted to descriptive statistics. Continuous variables were summarised using mean and standard deviation, or median and interquartile range (IQR), as appropriate, along with minimum and maximum values, and the 10th and 90th percentiles. In addition, TATs were categorized as less than 60 minutes, 60-90 minutes, and greater than 90 minutes for the total and analytical TAT. Given the lack of uniformity in the literature regarding preferred indicators for TAT reporting, multiple descriptive measures were presented to provide a comprehensive characterisation of TAT performance.

For the secondary objectives, data were collected to assess factors influencing TAT in outpatient and inpatient settings, and to evaluate staff awareness regarding TAT. Structured questionnaires were developed following an extensive review of published literature and expert consultation to identify workflow- and system-related factors potentially affecting TAT. Separate questionnaires were designed for outpatient attendees and inpatient healthcare personnel to ensure contextual relevance. In the outpatient setting, the initial questionnaire was pilot-tested among a small group of patients, which revealed that several literature-derived items were not comprehensible to lay respondents; consequently, the wording was simplified, and certain items were modified or omitted to improve clarity. The finalised outpatient questionnaire was translated into the local language and back-translated into English to ensure conceptual equivalence, and face validity was assessed with respect to clarity, comprehensibility, and content appropriateness. Patients were recruited by convenience sampling from the OPD waiting areas of the respective departments during data collection periods.

In the inpatient setting, data were collected through structured face-to-face interviews using a convenience sampling approach. Participants included five junior residents (one from each participating department), 25 nursing officers (five from each ward across five departments), and 10 health assistants (two from each of five departments) involved in test requisition, sample handling, and transport. In addition, one faculty member from each participating department was included to obtain supervisory perspectives. Staff awareness regarding TAT was assessed among central laboratory technicians using a structured five-item self-administered questionnaire, developed through expert consultation involving faculty from biochemistry, pathology, hospital administration, and the central laboratory, utilising a universal sampling technique. This questionnaire was pilot-tested to assess clarity and face validity, and participants answering at least three out of five items correctly were classified as “aware.” No formal content validity, construct validity, or reliability testing was undertaken, as the instrument was intended for operational assessment within the scope of this descriptive study. Data for all secondary objectives were collected prospectively through voluntary participation, and detailed descriptions of the questionnaires and assessed domains are provided in Appendix C.

The study received approval from the Institute Research Committee (IRC) (Ref. no.: AIIMS/Pat/IRC/PGTh/Jan21/15) and the Institute Ethics Committee (IEC) (Ref. no.: AIIMS/Pat/IEC/PGTh/Jan21/15). As the primary objective involved secondary analysis of routinely collected operational data and posed no risk to patients, the requirement for individual patient consent was waived by the IEC. Written informed consent was obtained exclusively for prospective, questionnaire-based components conducted under the secondary objectives, including assessment of factors influencing TAT and staff awareness. Participation in these components was voluntary, and confidentiality of all responses was maintained.

## Results

During data cleaning, a substantial proportion of records retrieved from the HIS were excluded due to incomplete or implausible time-stamp values, primarily arising from negative time intervals that are not logically compatible with TAT measurements. Of the 463,506 biochemical test records initially retrieved from the HIS (Table 1), 178,596 records (38.5%) contained complete and valid timestamp data and were included in the final analysis. Similarly, among 58,316 haematological test records retrieved (Table 1), 22,956 records (39.4%) were available for analysis after exclusion of invalid entries. The excluded records predominantly reflected inconsistencies in system-generated time stamps, rather than missing laboratory processes, and were therefore omitted to preserve the chronological coherence of TAT estimation.

**Table 1:** Total biochemistry and haematology test samples included N' = Total data retrieved; N = Actual data included LFT, liver function tests; TFT, thyroid function test; KFT, kidney function tests; RBS, random blood glucose; CBC, complete blood count; ESR, erythrocyte sedimentation rate; PT INR, prothrombin time-international normalized ratio; aPTT, activated partial thromboplastin time; HbA1c, glycated haemoglobin

Test Name	Biochemistry		Test Name	Haematology	
	N’	N		N’	N
LFT	223,694	84,526	CBC	37,441	15,595
KFT	219,009	88,892	ESR	4,664	1,345
TFT	9,817	2,141	PT INR	12,709	4,593
RBS	8,306	2,544	aPTT	3,502	1,423
HbA1C	2,680	493			
Total	463,506	178,596	Total	58,316	22,956

Table 2 presents the mean, median, and IQR values for the TAT components, in minutes, for biochemical and haematological investigations. However, for brevity, we focus solely on median values, which are commonly discussed in the literature and seem statistically appropriate. Comparing medians, biochemistry generally displayed shorter TAT durations than haematology, except for the analytical TAT, where the median for biochemistry samples (64 minutes) was slightly higher than that for haematology samples (38 minutes). A detailed descriptive analysis of TAT components for each test is provided in Appendix D for reference.

**Table 2:** Descriptive analysis of TAT components (N = 201,552) *all in minutes TAT, turnaround time; IQR, interquartile range

TAT Components	Biochemistry (N = 178,596)		Haematology (N = 22,956)	
	Mean (SD)	Median (IQR)	Mean (SD)	Median (IQR)
Pre-analytical TAT*	186 (163)	149 (104 - 218)	183 (213)	142 (98 - 203)
Analytical TAT*	97 (298)	64 (44.4 - 89)	82 (226)	38 (21 - 67)
Post-analytical TAT*	98.3 (304)	26 (9 - 64.4)	131 (454)	44 (18 - 102)

Table 3 shows that the median TAT for all samples was 4 hours and 38 minutes (278 minutes). For biochemistry, it was 4 hours and 39 minutes (279 minutes), and for haematology, it was 4 hours and 33 minutes (273 minutes). This means that biochemistry had a slightly longer median TAT than haematology. The 10th percentile represents the time below which 10% of the TAT values lie, while the 90th percentile represents the time below which 90% of the TAT values fall. From the same table, it is evident that 90% of biochemical test samples were completed within 2 hours and 36 minutes (156 minutes), while the same was achieved within 2 hours and 21 minutes (141 minutes) for haematology test samples. A detailed descriptive analysis of the TAT for each test is provided in Appendix D for reference.

**Table 3:** Descriptive analysis of total TAT (N = 201,552) *all in minutes TAT, turnaround time

Measures	Total TAT		
	Overall (N = 201,552)	Biochemistry (N = 178,596)	Haematology (N = 22,956)
Mean (SD)*	389 (462)	388 (451)	395 (539)
Median (Percentiles)*	278 (202 - 388)	279 (203 - 387)	273 (193 - 391)
10th Percentile*	154	156	141
90th Percentile*	572	573	564

**Figure 3:**
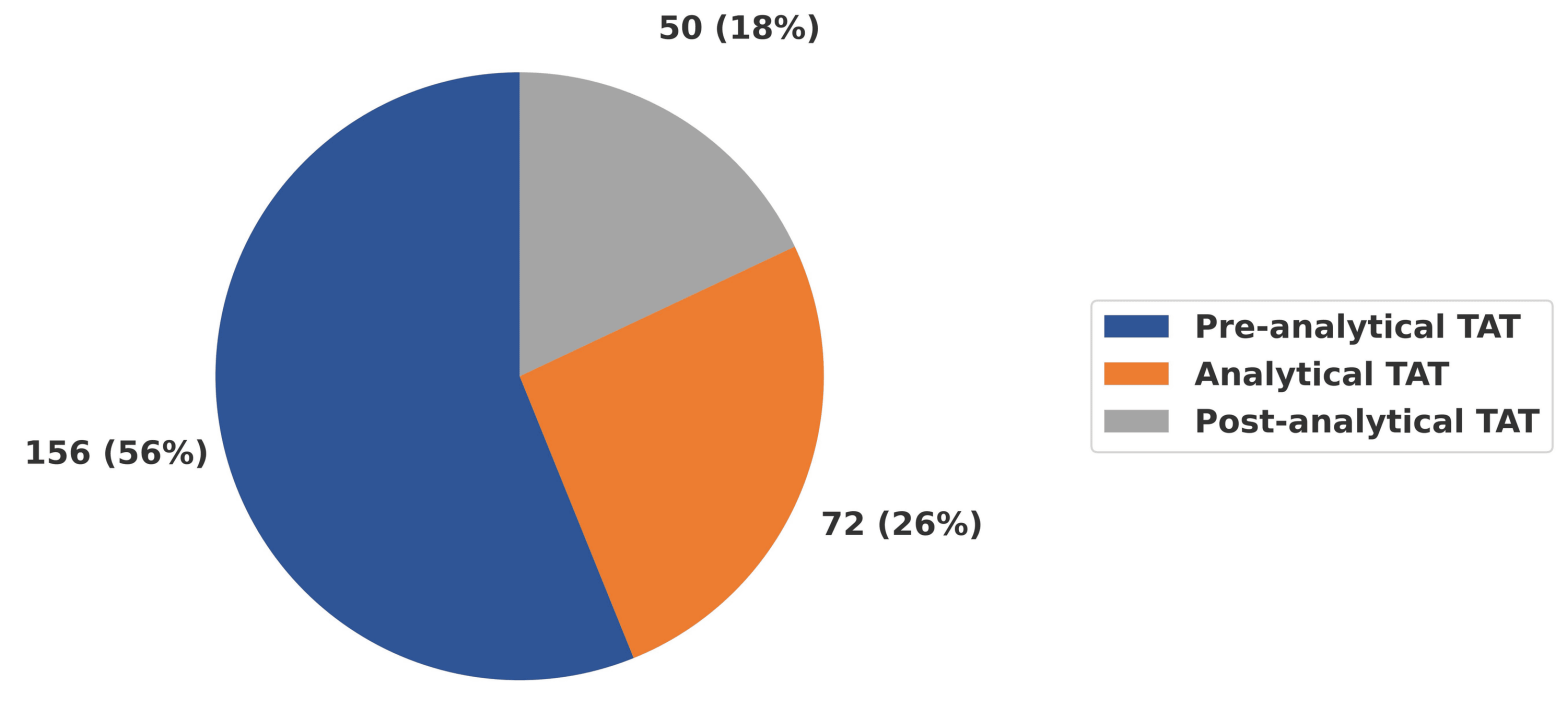
Percentage share of TAT components (overall total TAT = 278 minutes) Values represent the proportional distribution of the overall median total TAT (278 minutes). Pre-analytical TAT = 156 minutes (56%); analytical TAT = 72 minutes (26%); post-analytical TAT = 50 minutes (18%). Total samples included = 201,552. Unit: minutes. TAT, turnaround time

Figure 3 illustrates the proportional distribution of the median TAT (TAT = 278 minutes) across its components. The pre-analytical phase contributed the largest share, accounting for 56% (156 minutes) of the overall TAT. The analytical phase constituted 26% (72 minutes), while the post-analytical phase contributed the remaining 18% (50 minutes). These findings highlight that, although analytical processes represent core laboratory functions, delays occurring before and after the analytical stage together make up nearly half of the overall TAT.

**Figure 4:**
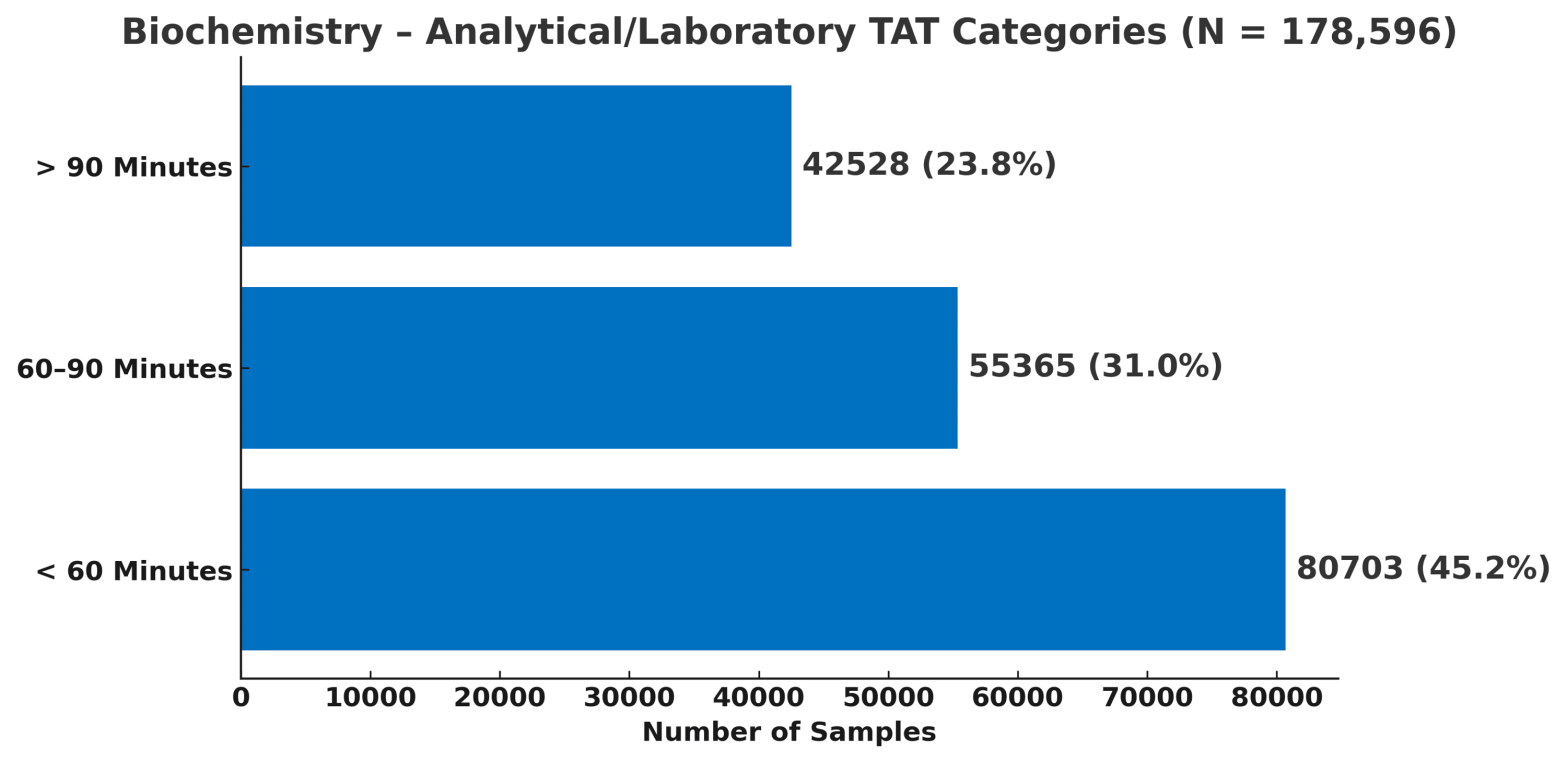
Distribution of biochemistry test samples according to their analytical TAT categories (N = 178,596) TAT, turnaround time

**Figure 5:**
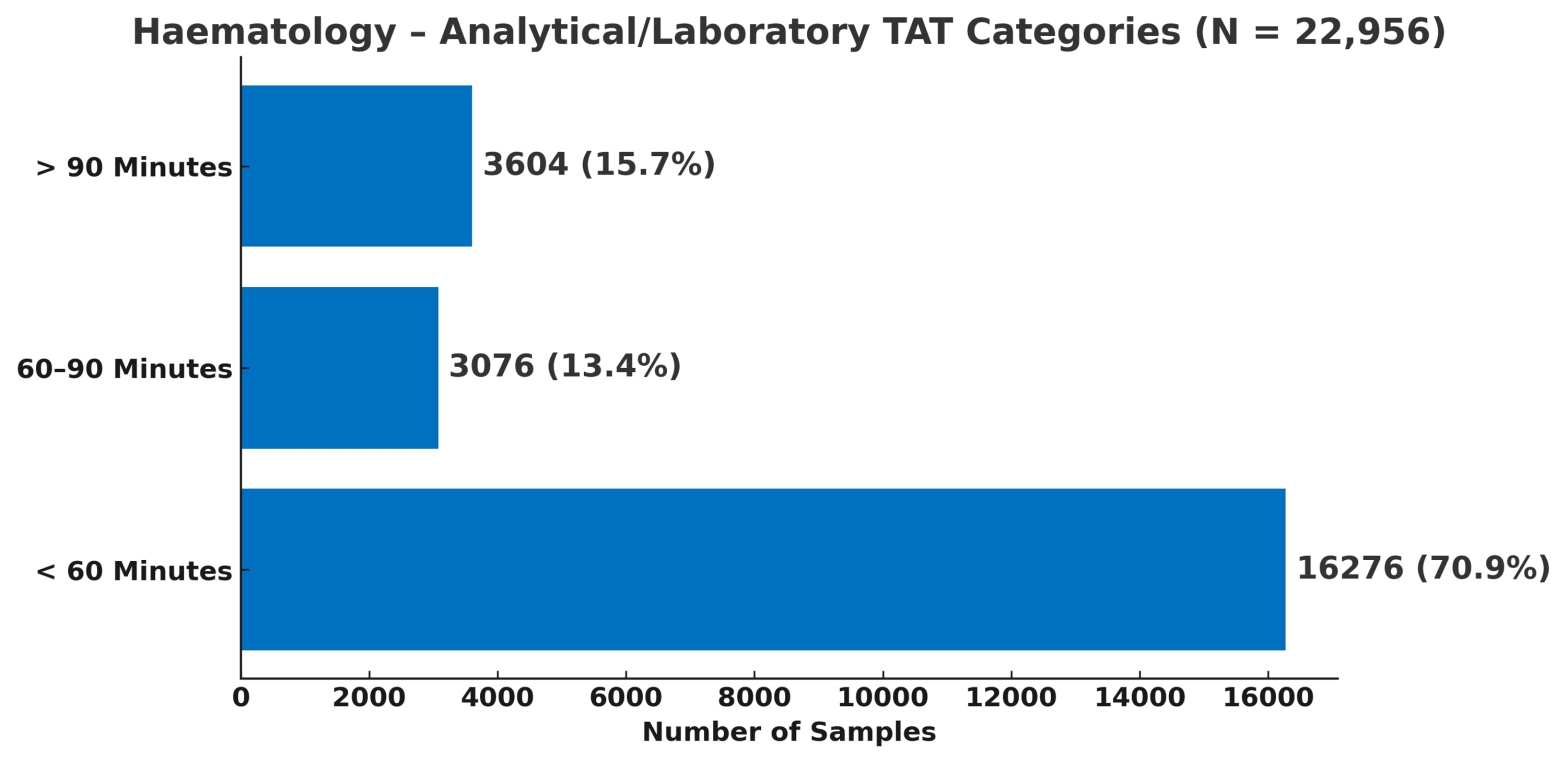
Distribution of haematology test samples according to their analytical TAT categories (N = 22,956) TAT, turnaround time

In biochemistry, 45.2% of samples (80,703/178,596) completed analytical processing within 60 minutes, whereas in haematology, a substantially higher proportion - 70.9% of samples (16,283/22,956) - met this benchmark, as shown in Figure 4 and Figure 5, respectively.

Table 4 displays the factors that OPD patients reported as influencing laboratory TAT. Responses were obtained from a total of 240 patients attending five departments: general medicine (n = 68), orthopaedics (n = 54), general surgery (n = 47), paediatrics (n = 43), and obstetrics and gynaecology (n = 30). Data were collected using a structured questionnaire, administered through face-to-face interviews in the outpatient waiting areas. Each factor is expressed as the proportion of respondents who reported its occurrence during the testing process.

**Table 4:** Factors affecting TAT in OPD setting as reported by patients (N = 240) TAT, turnaround time; OPD, outpatient department

Sl. No	Factors	Count (%)
1	Long waiting in the billing area	226 (94.2)
2	Long waiting in sample collection area	198 (82.6)
3	Long waiting for vacutainer collection	183 (76.2)
4	Insufficient manpower in the billing area	165 (68.6)
5	The billing and sample collection areas are far apart from each other	159 (66.3)
6	Insufficient manpower in the sample collection area	152 (63.4)
7	Patient misdirected to the wrong place/lack of signboards	148 (61.6)
8	Staff negligence (billing area, sample collection area, laboratory)	123 (51.2)
9	Payment-related issues	121 (50.6)
10	Insufficient manpower in the laboratory	115 (48)
11	Server, electricity, and network issues of billing system	114 (47.6)
12	Need for resampling (sample not sufficient or wasted)	91 (38)
13	Misplaced/lost samples during transport to laboratory	86 (36)
14	Computer/printer breakdown in report printing area	84 (35)
15	Computer/printer breakdown in billing area	78 (32.6)
16	Need for resampling (indeterminate results)	67 (28)
17	Patient name or investigation code misprinted in the bill	61 (25.6)
18	Equipment breakdown in laboratory	61 (25.6)
19	Vacutainer dispenser failure	56 (23.3)
20	Reports mismatched	56 (23.3)
21	Misprinted barcode on vacutainer	34 (14)

The distribution of factors reported by inpatient healthcare personnel (N = 45), as contributing to delays in TAT across the pre-analytical, analytical, and post-analytical phases, is summarised in Table 5.

**Table 5:** Factors affecting TAT in the inpatient setting as reported by healthcare workers (N = 45) TAT, turnaround time; HIS, hospital information system

Phase of TAT	Sl. No	Factor	Count (%)
Pre-analytical phase	1	Server, electricity and network issues of billing and HIS systems	37 (82.6)
	2	Transport from ward to central laboratory	36 (80.4)
	3	Haemolysis and need for repeat sampling	32 (70.0)
	4	Waiting at billing area	30 (67.0)
	5	Patient/attendant misdirected to wrong place	25 (56.4)
	6	Missing or incomplete payment	24 (54.3)
	7	Pre-analytical processing delays	18 (39.0)
	8	Incorrect entry of patient name or investigation code	17 (37.0)
	9	Waiting at vacutainer dispensing area	15 (33.0)
	10	Wrong payment not corresponding to prescribed tests	12 (26.1)
	11	Pneumatic tube breakdown	8 (17.4)
Analytical phase	12	Delay in analyte testing due to heavy workload	37 (82.6)
	13	Specimens misplaced in the laboratory	28 (63.0)
	14	Reagents out of stock/not supplied/expired	27 (61.0)
	15	Need for specimen dilution and re-run	21 (46.0)
	16	Equipment breakdown	19 (41.3)
Post-analytical phase	17	Delay in transcription from equipment to register and HIS	34 (76.0)
	18	Reporting system (HIS) downtime	28 (63.0)
	19	Delay in result validation	23 (50.0)
	20	Mismatch of reports in HIS	11 (24.0)

**Figure 6:**
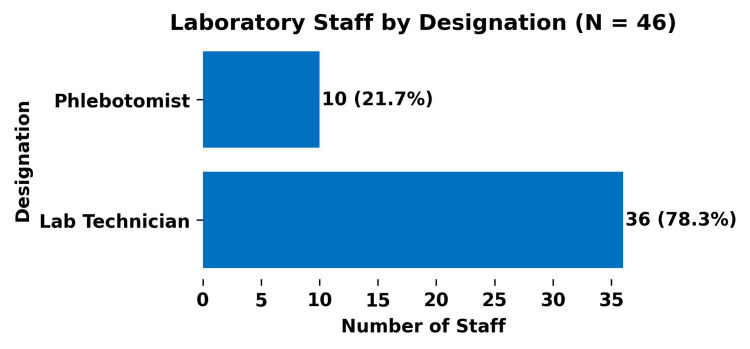
Laboratory staff designation (N = 46)

Among the 46 laboratory staff members, the distribution by designation was as follows (Figure 6): laboratory technicians constituted 36 (78.3%), and phlebotomists constituted 10 (21.7%). Although designated differently, both groups held the same educational qualification, a B.Sc. in Medical Laboratory Technology (MLT).

**Figure 7:**
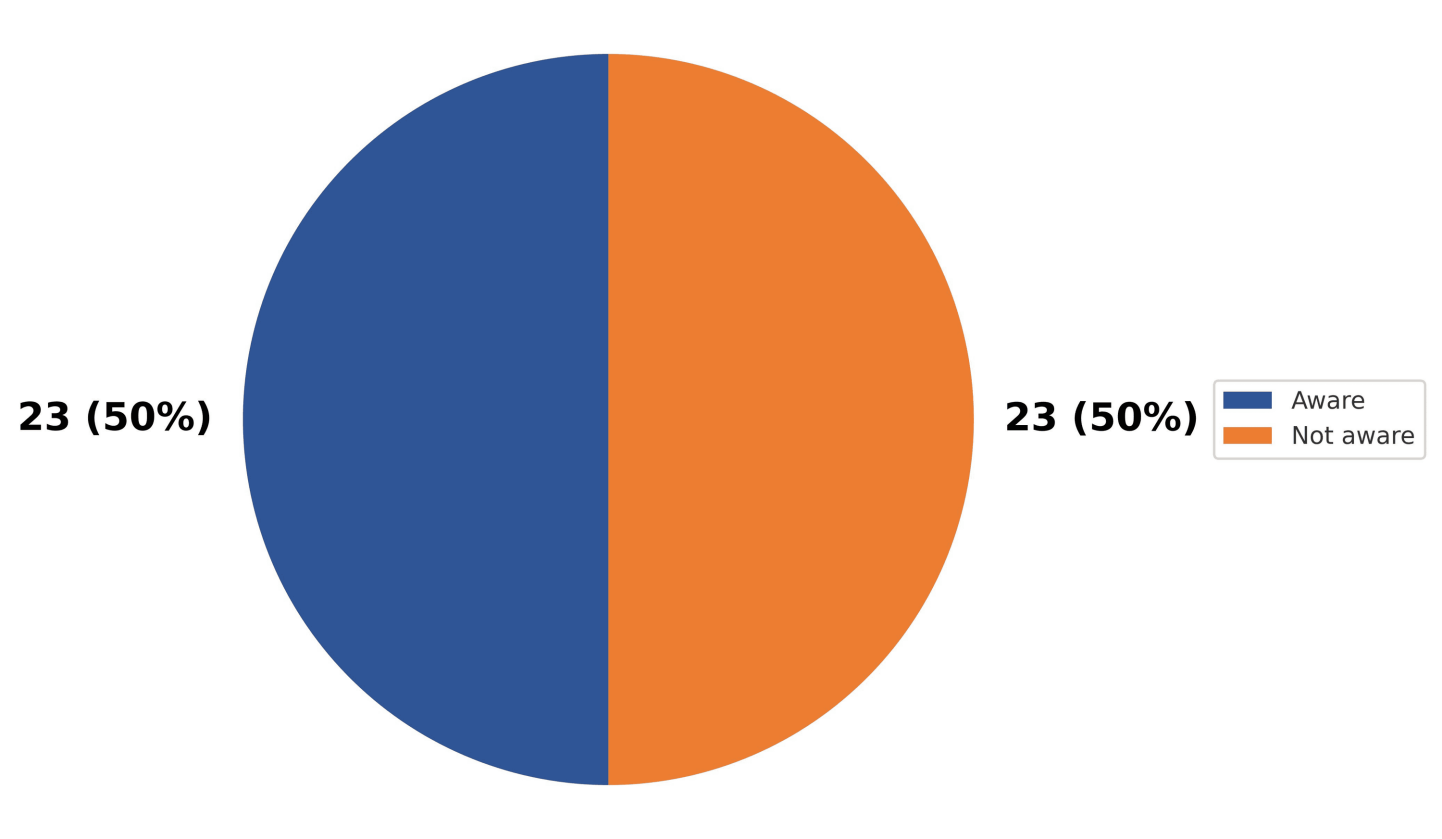
Proportion of laboratory staff aware of TAT (N = 46) TAT, turnaround time

The laboratory staff had an average of 11.2 years of experience (SD = 3.5), with a range from 4 to 18 years. Only half of the laboratory staff was aware of the TAT, as shown in Figure 7.

## Discussion

Institutional workflow design, automation, staffing patterns, patient volume, and information system maturity inherently influence TAT, limiting its generalisability across healthcare settings. In line with ISO 15189:2012, the present study evaluated TAT as an internal quality-assurance exercise, to establish baseline performance metrics within a high-volume tertiary-care government hospital. Selected studies were cited only to contextualise observed patterns and systemic contributors to the delay.

A major operational observation was the limited availability of valid HIS timestamp data, with only 38.6% of generated samples eligible for analysis, due to implausible or inconsistent timestamps. Institutional laboratory audits have also reported similar problems with reliable time capture. In these cases, digital system instability makes it challenging to evaluate performance, even when services are being delivered on a regular basis. Wankar [21] highlighted the dependence of meaningful TAT assessment on accurate timestamp recording. Although the exclusion of invalid records may influence absolute TAT values, the internally consistent, phase-wise patterns observed in this study are unlikely to be artefacts, as analyses were restricted to chronologically coherent records. Notably, the magnitude of unusable data itself represents an important quality signal, underscoring the critical role of HIS robustness in laboratory performance monitoring.

Phase-wise analysis showed that non-analytical processes dominated total TAT. The pre-analytical phase accounted for approximately 56% of total TAT, while the post-analytical phase contributed an additional 18%. Manor [11] and Rollo and Fauser [22] similarly identified non-analytical phases as major determinants of prolonged TAT, though their proportional contributions were lower than those observed in the present study, where non-analytical processes, together, constituted approximately 74% of total TAT. In contrast, Chung et al. [15] reported substantially lower contributions from pre-analytical and post-analytical phases, likely reflecting differences in automation, staffing ratios, workflow integration, and patient throughput. Despite numerical variability, the consistent interpretation across studies is that inefficiencies outside the analytical core disproportionately prolong overall TAT.

The factor analysis offered theoretical explanations for these findings. In the outpatient setting, patients most frequently reported prolonged waiting in the billing, vacutainer distribution, and sample collection areas. These delays reflect the operational realities of government hospitals, with daily outpatient footfall exceeding 2,000 patients, particularly during special service days such as Pradhan Mantri Surakshit Matritva Abhiyan, when billing wait times frequently extend to 20-30 minutes. Comparable crowd- and workflow-related pre-analytical delays have been reported by Belay and Deress [23], and Shiferaw and Yismaw [24]. In the inpatient setting, healthcare personnel identified transport delays, haemolysis requiring repeat sampling, and HIS or network disruptions as key contributors to pre-analytical delay, consistent with observations by Mutema et al. [25] and Mwogi et al. [26]. The concordance between patient-reported and staff-reported factors indicates that delays are predominantly structural rather than sporadic.

Analytical performance, when evaluated independently, was comparatively efficient, particularly for haematology. Analytical TAT was categorised as <60 minutes, 60-90 minutes, and >90 minutes, based on established laboratory performance benchmarks. Hawkins [5] proposed analytical TAT targets of ≤60 minutes for haematology and ≤90 minutes for biochemistry. Accordingly, the <60-minute category represents optimal performance, 60-90 minutes acceptable but borderline performance, and >90 minutes delayed processing. When these benchmarks were applied to the total TAT, only 0.7% of biochemistry samples and 0.5% of haematology samples met the target, highlighting the impracticality of applying analytical benchmarks to the entire testing cycle in high-volume public institutions. When applied exclusively to the analytical phase, however, 76% of biochemistry samples and 71% of haematology samples met acceptable performance standards, confirming that analytical processes were largely compliant once non-analytical delays were excluded.

Post-analytical delays in this study were primarily attributable to result validation by postgraduate trainees and senior residents, balancing clinical and academic responsibilities, despite timely result generation. Post-analytical delays related to result verification and reporting workflows have also been described by Jalili et al. [10] and Alain et al. [27], although the specific validation mechanisms and staffing structures differed across institutions.

Another important factor in improving performance is that laboratory staff know about TAT. In our study, only 50% of staff demonstrated adequate awareness of TAT requirements, a figure lower than the knowledge (60%), attitude (86%), and practice (63%) scores reported by Gebreyes et al. [28]. While methodological differences may partly explain this discrepancy, it highlights an important human-factor component, which requires targeted training and sensitisation.

This study offers a thorough evaluation of TAT for standard biochemical and haematological tests in a high-volume tertiary-care facility. By analysing phase-specific delays, the study offers a more granular understanding of workflow inefficiencies than traditional, single-parameter evaluations. The large retrospective dataset, spanning an extended timeframe, enabled robust trend analysis without disrupting laboratory operations. Use of median and IQR statistics provided an accurate representation of TAT distributions in the presence of non-normality and operational variability. The inclusion of diverse patient categories, such as outpatient, inpatient, and emergency, allowed us to capture systemwide patterns of TAT behaviour, strengthening the interpretability and applicability of findings across service areas. An examination of delays across the pre-analytical, analytical, and post-analytical domains helped identify specific bottlenecks, which presented tangible opportunities for workflow optimisation.

Despite the strengths of this comprehensive, phase-wise evaluation, several limitations merit consideration. First, TAT assessment is inherently context-dependent, as institutional workflow design, degree of automation, staffing patterns, patient volume, and information system maturity vary widely across healthcare settings; consequently, the findings are primarily applicable to similar high-volume, tertiary-care public hospitals. Second, a substantial proportion of laboratory records could not be analysed because of inconsistencies and implausible values in the system-generated HIS time stamps, resulting in the exclusion of a large fraction of retrieved data. This data loss may have introduced selection bias if timestamp errors were not randomly distributed across tests or workflow stages; however, the exclusion of such records was necessary to preserve chronological validity and interpretability of TAT estimates. Importantly, the magnitude of unusable data itself highlights a critical system-level limitation, underscoring the dependence of reliable TAT monitoring on robust digital infrastructure. Third, the HIS did not permit differentiation between outpatient, inpatient, and emergency samples at the individual record level, limiting stratified TAT analysis by care setting. Fourth, reliance on electronic time stamps precluded assessment of patient-related delays occurring before formal test requisition or sample registration, potentially underestimating certain pre-analytical contributors. Fifth, the staff awareness assessment was based on a short questionnaire that was only tested for face validity, not for content validity, construct validity, or reliability, making it difficult to interpret the absolute awareness estimates. Finally, as a single-centre study, generalisability beyond the study institution remains limited. Nevertheless, these constraints reflect real-world operational challenges in public-sector laboratories and do not detract from the study’s value as an institutional quality-assurance exercise aimed at identifying actionable workflow bottlenecks and informing system-level improvement.

Several operational contributors to TAT delays were identified across the testing cycle. Prolonged waiting in billing, vacutainer distribution, and sample collection areas, along with the physical separation between billing counters and collection points, substantially prolonged pre-analytical timelines, particularly under conditions of high outpatient footfall. Patient misdirection further compounded these delays. In the inpatient setting, manual transport of samples from wards to the central laboratory, haemolysis necessitating repeat sampling, and intermittent HIS or network disruptions emerged as prominent contributors. Within the laboratory, heavy workload occasionally delayed analyte processing, while manual transcription of results from instruments to registers, and subsequently into the HIS, introduced avoidable bottlenecks. Post-analytical delays were further influenced by result validation responsibilities borne by postgraduate trainees and senior residents, alongside competing clinical and academic demands.

Addressing these challenges requires coordinated, system-level reform. Strengthening the HIS through improved stratification at data origin points, long-term data preservation, routine audit-based quality assurance, and timely software maintenance would enhance workflow traceability and reliability. Managing high patient volumes necessitates expansion of billing infrastructure, adoption of online billing platforms, increased availability of vacutainer dispensers, improved signage, and workforce augmentation in billing and sample-collection areas. Establishing dedicated pneumatic transport for inpatient samples and reducing physical separation between billing and collection areas would further minimise transport-related delays. Within the laboratory, enforcing stricter time management for manual tests, enhancing staff competency through targeted training, streamlining validation through automated alerts, and instituting priority pathways for urgent samples are essential. Incorporation of point-of-care testing in emergency settings, and sustained educational initiatives on TAT significance, would further strengthen patient care delivery and institutional efficiency.

## Conclusions

This study evaluated laboratory efficiency through the assessment of TAT for routine biochemical and haematological tests at AIIMS Patna. While a substantial proportion of samples met established analytical performance benchmarks, the findings emphasise that it is necessary to address persistent delays arising outside the analytical phase. The study also revealed that limitations in the HIS, particularly inconsistencies in time-stamp recording, constrained the completeness of available data, and reaffirmed the need for reliable digital infrastructure for accurate performance monitoring. By establishing baseline TAT metrics and identifying key workflow bottlenecks, this work provides a pragmatic framework for targeted quality-improvement initiatives. Strengthening data capture systems, reinforcing staff training, and optimising operational processes across the testing cycle are essential to maintaining efficiency gains and enhancing timely diagnostic service delivery in high-volume, public healthcare settings.

## Appendices

Appendix A

Laboratory Investigations Included in the Study as per the Expert Advice From the Respective Departments

The tests included in this study were those identified by an expert panel from the respective departments as representative of routine diagnostic workload and essential for assessing laboratory efficiency. For biochemistry, the selected tests comprised liver function tests (LFT), kidney function tests (KFT), thyroid profiles, random blood glucose (RBS), and HbA1c. For haematology, the expert panel recommended inclusion of Complete Blood Count (CBC), Erythrocyte Sedimentation Rate (ESR), Prothrombin Time (PT) with INR, and Activated Partial Thromboplastin Time (aPTT).

Appendix B

Activities In Between the Time Stamps in the Case of the OPD Setting

In the OPD setting, the interval between test requisition and sample collection included several steps: waiting at the billing counter, the time taken for the patient or attendant to reach the central sample collection area in the OPD basement, waiting for a token, dispersing barcoded vacutainers, and performing the subsequent phlebotomy. Once collected, samples were accumulated until a sufficient batch was available for generating a packing list, after which they were transported together through the pneumatic tubing system to the central laboratory. The period from packing-list generation to sample acceptance included physical transport via the pneumatic tube and the verification of specimen adequacy upon arrival; samples not meeting quality standards required recollection. The analytical phase, from sample acceptance to result entry, comprised instrument run time and the manual transcription of analyser outputs into the HIS. Following results entry, postgraduate trainees (JRs) validated the reports, making them available to patients.

Activities In Between the Time Stamps in the Case of the IPD Setting

In the IPD setting, requisition and sample collection were performed within the ward by nursing officers. Although financial clearance was generally pre-existing through the patient’s AIIMS account, either deposited by the patient for APL status or by the government for BPL beneficiaries, the attendant was still required to complete an IPD billing step. Unlike OPD procedures, IPD samples did not use barcoded Vacutainers; instead, nursing officers manually labelled tubes with patient details. Samples collected from each ward were pooled before transport, with health attendants (HAs) delivering them to the central laboratory. The same workflow as in the OPD setting was followed by subsequent processes, that is, from sample acceptance to result entry and from result entry to validation.

Appendix C

Laboratory Staff Awareness

The questions evaluated conceptual understanding of TAT (“How would you define turnaround time (TAT)?”), its clinical relevance (“Why is TAT important?”), identification of the most critical component of TAT in laboratory performance (“Which part of TAT is most crucial in assessing laboratory efficiency?”), recognition of system-level contributors (“Can you enumerate any two factors that could impact TAT?”) and awareness of institutional measures to address delays (“In the context of our hospital laboratory, can you provide an example of a strategy or initiative implemented to address TAT challenges?”). The draft questionnaire underwent pilot testing to ensure face validity and clarity; however, no formal content or construct validation was performed due to the operational nature of the assessment. Each correct response was scored as one point, and staff answering at least three of the five questions correctly were categorised as “aware.”

Appendix D

Turnaround Time (TAT) Summary of Individual Tests

**Table 6:** Descriptive statistics of turnaround time (TAT) components for routine haematology and biochemistry investigations included *all values in minutes

Investigation	Pre-analytical TAT*		Analytical TAT*		Post-analytical TAT*	
	Mean (SD)	Median (IQR)	Mean (SD)	Median (IQR)	Mean (SD)	Median (IQR)
Liver Function Test (LFT)	183 (158)	148 (104–212)	88 (274)	63.3 (44–88.2)	104 (304)	27.3 (9.1–67.2)
Kidney Function Test (KFT)	190 (156)	151 (104–230)	99 (278)	65 (46–89.3)	96 (310)	26 (9–63.2)
Thyroid Function Test (TFT)	172 (357)	144 (116–177)	67 (109)	49.4 (35.4–67.1)	52 (147)	20.5 (8–49.3)
Random Blood Sugar (RBS)	164 (204)	149 (115–188)	63.3 (125)	45 (32–67.3)	39 (122)	12 (5–32)
Glycosylated Haemoglobin (HbA1c)	288 (450)	151 (106–246)	1623 (1690)	1745 (246–1888)	53.6 (288)	31.5 (5–32)
Complete Blood Count (CBC)	180 (202)	140 (97–202)	63.3 (198)	31 (17.2–52.4)	120 (275)	47 (21–109)
Erythrocyte Sedimentation Rate (ESR)	227 (331)	158 (114–217)	69 (108)	47 (27–84)	125 (314)	48 (21.2–97.3)
Prothrombin Time INR (PT-INR)	180 (202)	140 (97–202)	63.3 (198)	31 (17.2–52.4)	120 (275)	47 (21–109)
Activated Partial Thromboplastin Time (aPTT)	178 (193)	144 (98.4–201)	131 (295)	65 (39.4–101)	159 (832)	35 (14–84)

**Table 7:** Descriptive summary of total turnaround time (TAT) across investigations included *all in minutes

Investigation	Mean (SD)*	Median (IQR)*	10th Percentile*	90th Percentile*
Liver Function Test (LFT)	389 (445)	278 (202–383)	156	574
Kidney Function Test (KFT)	384 (429)	283 (205–395)	156	571
Thyroid Function Test (TFT)	259 (405)	205 (162–256)	137	332
Random Blood Sugar (RBS)	266 (264)	230 (182–288)	151	352
Glycosylated Haemoglobin (HbA1c)	1965 (1727)	1956 (1504–2066)	319	3402
Complete Blood Count (CBC)	364 (383)	263 (183–385)	134	535
Erythrocyte Sedimentation Rate (ESR)	420 (459)	296 (225–385)	170	548
Prothrombin Time – INR (PT-INR)	364 (383)	263 (183–385)	134	535
Activated Partial Thromboplastin Time (aPTT)	468 (890)	290 (215–407)	160	1572
